# Co-design values: public health perspectives

**DOI:** 10.3389/fpubh.2025.1706178

**Published:** 2026-01-09

**Authors:** Sierra Allen, Xandria Hair, Ed Miranda, Laura Jean Brennan, Lena Hatchett

**Affiliations:** 1 Cook County Department of Public Health, Forest Park, IL, United States; 2 Community Health and Development Contractor, Portland, OR, United States; 3 Loyola University Chicago, Chicago, IL, United States

**Keywords:** public health, co-design, health equity, public health professionals, socioecological model

## Abstract

The report describes the Cook County Department of Public Health (CCDPH) Co-design Values Model from the perspective of public health professionals. The goal of the model is to improve health equity through community collaboration. The study utilized multiple data sources over 26 months (May 2023–July 2025), including progress reports, meeting notes, and data summaries from 36 community-based organizations, to analyze qualitative and quantitative insights from the Regional Learning and Action Network Co-Design Team (RCT). The co-design process revealed an effective approach toward increased learning and capacity among public health professionals. The Co-design Values Model highlights the importance of valuing lived experiences (individual level), navigating power dynamics (organizational level), and fostering collaboration (community level). The model offers a valuable framework for public health professionals to engage in authentic and equitable co-design approaches, building capacity, trust, and accountability to address health inequities.

## Introduction

Cook County Department of Public Health (CCDPH) is the state-certified public health agency serving suburban Cook County, Illinois, the second most populous county in the United States. As part of the Cook County Health system, CCDPH promotes and protects the health of approximately 2.5 million residents by addressing the social and structural determinants that shape population health in collaboration with local, state, and federal partners. During the COVID-19 pandemic, suburban Cook County (SCC) communities faced detrimental infection, hospitalization, and mortality rates. According to the 2020–2021 census data, predominantly Black, Hispanic, and immigrant communities experienced the highest disparities (1). These disparities were compounded by misinformation on social media, long-standing government mistrust, and shortages of public health professionals, all of which heighten public distrust in vaccines and prevention measures (2). In response to this climate of community distrust, CCDPH sought to rebuild partnerships by embedding co-design into its regional engagement strategy. This approach moved beyond traditional, top-down public health messaging and included resource co-creation and mutual decision-making (Lynch et al., 2025; Martin et al., 2023).

SCC is home to 5.28 million residents representing racial, cultural, and socioeconomic background diversity. The northern suburbs are predominantly Asian and White, with higher household incomes and access to healthcare resources, while the western suburbs are experiencing rapid growth among Hispanic and immigrant populations, many of whom face language and cultural barriers in navigating health systems. In contrast, the southern and southwest suburbs, largely Black communities, experience higher rates of poverty, chronic disease, and limited access to care (1). These regional contrasts reveal deep structural inequities that were further magnified during the pandemic. Recognizing that one approach could not address these diverse community realities, CCDPH pivoted and implemented a co-design framework that relied on trusted community partners, culturally responsive strategies, and continuous reflection guided by shared principles (see Appendix A). The co-design process is emerging as a key strategy to advance health equity [(3); Messiah et al., 2023]. CCDPH defined co-design as a collaborative appraoch that engaged community members, partners, and government entities directly in the decision-making process and emphasized sharing power, lived experiences, and health equity-driven solutions to achieve a common goal (McKercher, 2020) (4).

For the Regional Learning and Action Network (RLAN) Summits, the co-design approach ensured that the COVID-19 capacity-building activities were community-centered and responsive to the needs of the residents they serve. Initially launched virtually during the pandemic, RLAN sessions featured COVID-19 information updates, partner spotlights, and open question-and-answer forums, allowing community partners direct access to health experts. Over time, these sessions evolved into in-person convenings that reconnected separated regions, fostering collaboration, shared learning, and trust between public health professionals and community partners. The longer-term aim is to sustain relationships between public health professionals and community partners. This model underscores the essential role of authentic collaboration in closing health equity gaps, especially in communities historically marginalized or misrepresented within public health systems.

## Co-design to advance health equity

Public health literature highlights collaboration and co-design as a robust approach to advancing public health workforce development and promoting health equity within communities (5, 6). Across diverse contexts, co-design redistributes power, elevates community voices, and fosters authentic, trust-based relationships (7). Within CCDPH, co-design was intentionally embedded in pandemic response efforts to ensure that both professional expertise and community knowledge shaped decision-making. CCDPH positioned co-design not only as a method for collaboration but also as a pathway to equity, one that acknowledges historical inequities and empowers residents to define what health and well-being mean within their communities. When applied intentionally through inclusive collaboration and shared decision-making, co-design improves the public health workforce and strengthens community capacity. Below is a review of co-design studies and values that promote health equity.

## Co-design values model

This Co-design Values Model was adapted from the Social Ecological Model (8) to illustrate how (1) valuing lived experiences at the individual level, (2) financial and power dynamics at the organizational level, and (3) collaboration at the community level shape equitable collaboration. This conceptual model highlights the factors influencing co-design based on the experiences of public health professionals.

### Valuing lived experiences (individual level)

Co-designers were encouraged to set aside formal roles and engage based on lived experiences. This fostered authenticity, communication, and mutual learning, primarily through the contributions of community partners who shared insights from their organizational and personal perspectives. At the start of each meeting, co-designers ground themselves by identifying with a co-designed principle that resonated with them.

### Financial support and power dynamics (organizational level)

Funding sources are critical for supporting health equity coaches, community partners, public health professionals, and technical assistants. A few co-design studies mention the limitations and financial challenges of a co-design process (9–11). Power dynamics emerged early, prompting training to help CCDPH staff develop their skills. This was a challenge at the beginning of the journey. However, it stabilized with open dialogue and a fluid process, which reduced hierarchies and shared influence across the co-design team. A notable example involved community partners who challenged federal and local data systems that identified them as White in census reporting. Their advocacy led to recognition of Middle Eastern/North African (MENA) as identity categories, demonstrating how co-design principles can extend beyond program planning to influence structural systems of representation and equity. Their success revealed how redistributing power through co-design can influence not only program outcomes but also the systems that define who is seen and counted in public health.

### Collaboration (community level)

Effective co-design requires training and coaching to ensure community partners can fully participate. Each component of the summit is co-developed by at least one community partner, a public health professional, a health equity coach, and a technical assistant, ensuring equal ownership and contribution to the project. CCDPH staff flexibly adapted when capacity gaps arose, reinforcing trust and commitment. These practices help build a shared responsibility and ensure relationships are authentic and grounded in mutual respect. Efforts supported this level by co-designing breakout sessions at the RLAN summit to share and engage requested information to CBOs.

**Figure 1:**
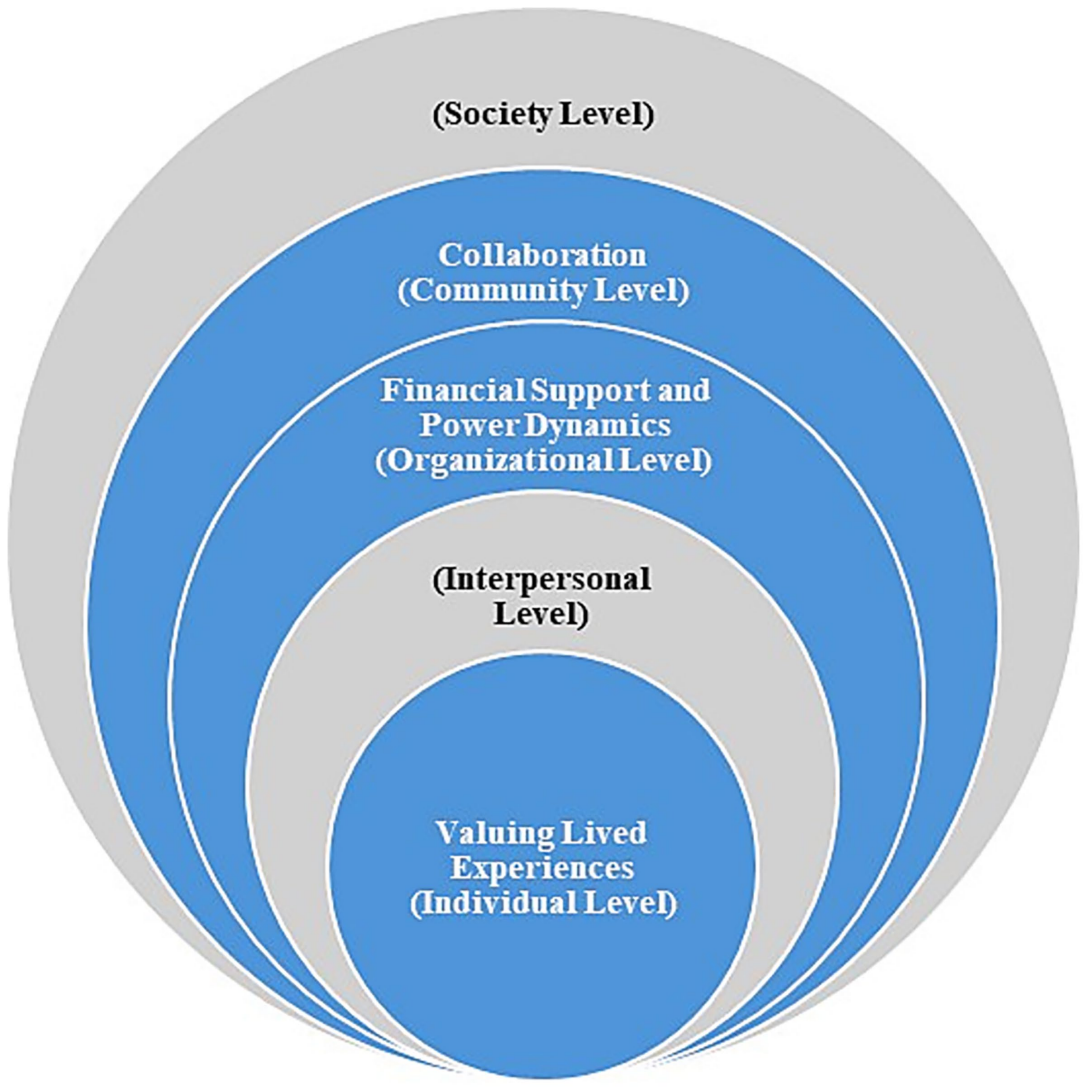
Co-design values model.

Figure 1 illustrates how CCDPH’s co-design process operates as an ecosystem, one in which reflection, shared power, and collaboration continually inform one another to sustain equitable public health practice.

### Valuing lived experiences

Meigs et al. (12) introduced nine community-led transformation (CLT) principles to restructure public health learning systems by embedding community-based organizations as equal partners. Centering community voices and fostering co-leadership; the CLT framework supports inclusive, responsive, and sustainable public health systems that advance health equity. Kia-Keating et al. (13) highlights the importance of relationship-centered and culturally responsive co-design processes, demonstrating how program development can be transformed through collaboration.

Valuing lived experiences was central to the co-design process at CCDPH. The perspectives of public health professionals, community partners, and health equity coaches each contributed insight into how equity could be operationalized within the communities they served, helping shape program design, outreach strategies, and the framing of public health messages. For instance, one community partner who organizes with Hispanic residents in the western suburbs leveraged their personal and cultural background to build trust among local families. Because they shared the same language, cultural references, and lived experiences as the community, residents were more willing to attend vaccine events, seek accurate information, and engage with CCDPH resources. In this model, lived experience operates not as anecdotal input but as a structural mechanism for redistributing power and reframing expertise within public health practices.

### Power dynamics in co-design

Farr (14) investigates the complex power structures within co-design and shows that while the process can challenge hierarchies, genuine power sharing requires continuous, inclusive reflection and dialogue.

Addressing power dynamics is foundational to advancing health equity. Redistributing decision-making authority within the RLAN co-design team (RCT) allows historically marginalized voices, particularly from Black, Hispanic, and immigrant communities in SCC, to influence programs that directly affect their health outcomes. Within CCDPH’s co-design process, this redistribution of power was not theoretical but intentionally practiced. Public health professionals shared facilitation, program design, and planning responsibilities with community partners throughout the RLAN development stages. For example, community partners co-led breakout sessions, shaped summit agendas, and co-authored communication materials distributed across suburban regions. This structure ensured that power was exercised collectively, rather than controlled by agency staff. By co-owning decision-making, RCT strengthened trust and accountability, two essential ingredients for equitable collaboration. This intentional rebalancing of power shifted relationships from transactional partnerships to transformative collaborations, fostering greater trust and ownership in public health outcomes (3).

Grindell et al. (15) conducted a review of 24 international studies, identifying mechanisms facilitating shared power: equal partners, valuing diverse knowledge, fostering trust, and ensuring shared ownership. Both articles identify co-design as a relationship-based practice centered on redistributing power.

### Co-design and collaboration

Benz et al. (16) implemented a community-based participatory research approach to co-design tele-practice services with individuals who have disabilities and providers, emphasizing small group open communication, accessibility, and skill-building. Debenham et al. (17) engaged with youth to co-design digital public health resources, resulting in relatable health content. Both studies underscore co-design as a collaborative approach that values the input of all stakeholders.

Within the RCT, collaboration was defined not just by inclusion but by responsiveness. During the planning of the RLAN summits, community partners identified that the registration materials and presentation formats were not fully accessible for individuals with disabilities and non-English-speaking participants. In response, the RCT co-created ADA-compliant meeting spaces and translated materials in Spanish and other prevelant languages across SCC. This process requires flexibility and humility, acknowledging that even well-intentioned planning can overlook accessibility needs. By acting on partner feedback, the RCT strengthened its collective capacity to create culturally and language-responsive environments. These changes not only enhanced participation but also demonstrated shared accountability, where both public health professionals and community partners worked together to remove barriers and model equity in practice.

## Methods

The CCDPH Co-Design Values Model captures the experience of the RCT. The RCT involved six community partners, six public health professionals, and four health equity coaches, who conducted the four phases of the RLAN summits. The development of the model was guided by community-based participatory research (CBPR) principles, emphasizing shared decision-making, reflection, and adaptation throughout the process (18).

The Co-Design Values Model was adapted from the Social Ecological Model (8) to capture how individual, organizational, and community-level factors interact to influence equitable engagement. This framework aligns co-design as a multilevel process defined by feedback loops of trust, power, and collaboration between public health institutions and community partners. Figure 1 illustrates how these levels, valuing lived experiences (individual), navigating power and funding structures (organizational), and fostering collaboration (community), connect within the co-design framework.

The planning phase of the summit was approximately 6 months. The implementation phase is ongoing, and this report captures efforts over 26 months. The RCT implemented a four-phase development process: Planning, Implementation, Facilitation, and Reflection, spanning from January 2020 to July 2025. The planning phase lasted approximately 6 months and focused on developing agendas and onboarding staff and partners through equity-centered training. The implementation and dissemination phases are ongoing, and this report captures the first 26 months of effort.

Data were collected and summarized over 26 months and included meeting notes and data summaries submitted by 36 community partners. These materials capture both qualitative reflections and quantitative indicators of the summit’s progress and engagement. Throughout implementation, RCT intentionally shared facilitation, planning, and design responsibilities to ensure equitable decision-making. Community partner reflections, surveys, and debriefs were conducted to capture emerging insights and challenges. For instance, an early RLAN survey revealed that many attendees were disappointed by the lack of coffee and tea after lunch, an unexpected and impactful finding that reminded the RCT of the importance of attending to everyday comforts as a form of care and hospitality. Such observations reinforced the value of continuous feedback and adaptive design as an essential component of equitable co-design.

The documentation developed and collected through these phases, meeting notes, case summaries, and partner reflections, served as the primary data sources for analysis. Qualitative data were analyzed to identify recurring themes related to trust, power-sharing, and collaboration, while quantitative measures like meeting attendance and participation frequency helped track engagement trends. The integration of qualitative and quantitative insights provided a comprehensive picture of how co-design values evolved throughout implementation. No human subject data were collected; all insights were drawn from organizational documentation and partner reflections. While no formal human subjects research was conducted, qualitative insights were obtained with institutional permission, in alignment with ethical standards for participatory evaluation.

**Figure 2:**
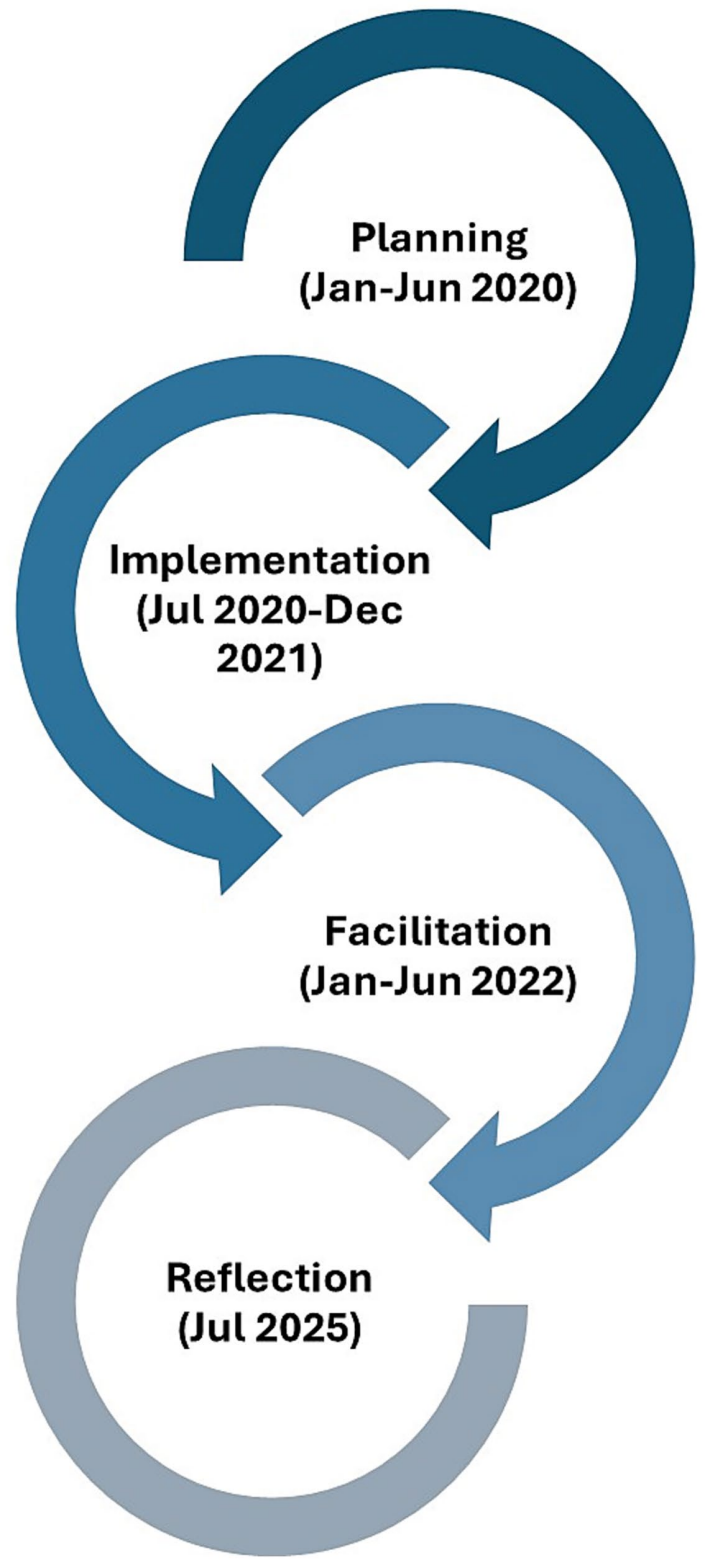
Co-design phases.

The timeline of the co-design process is illustrated in Figure 2, which shows how activities overlapped across four phases: Planning (Jan–Jun 2020), Implementation (Jul 2020–Dec 2021), Facilitation (Jan–Jun 2022), Reflection (Jul 2025). This cycle of progress demonstrates how feedback from each phase directly informed the next, reinforcing a culture of learning, adaptation, and collaboration among all co-design participants.

## Results

Many traditional public health strategies follow linear timelines, typically ranging from 3 to 12 months. However, our co-design process revealed iterative thinking and concentric circles. As we experienced, dimensions of co-design, learning, and capacity increased among public health professionals. We adopted the human-centered design framework (19) to depict the co-design experiences over time, as illustrated in Figure 1. Analysis of meeting summaries, partner reflections, and surveys identified three overarching themes: (1) professional learning and adaptive practice, (2) trust and relationship building, and (3) collaboration and shared accountability.

### Professional learning and adaptive practice

Public health professionals involved in the RLAN demonstrated increased confidence and flexibility in co-design facilitation over time. Early documentation reflected uncertainty around how to balance community leadership with agency responsibilities; however, repeated engagement with community partners helped clarify their roles. The introduction of health equity coaches was particularly effective in modeling reflective practice by helping staff recognize the importance of slowing down, listening, and acknowledging discomfort as a part of growth. This practice reflects the co-design principle of practicing the art of being comfortable while being uncomfortable. Through these experiences, public health professionals’ learning shifted from technical skill-building to a mindset of adaptive leadership, grounded in humility and shared ownership.

### Trust and relationship building

Trust emerged as both a process and a product of the co-design experience. Partners consistently noted that CCDPH’s willingness to share decision-making and visibly act on feedback strengthened credibility and confidence. This aligns with findings from Jagosh et al. (20), whose realist evaluation of community-based participatory research demonstrates that trust-building is an outcome of equitable partnerships, that strengthen long-term collaboration beyond the initial project scope. For example, when participants highlighted gaps during RLAN planning, the RCT co-created ADA-compliant spaces, and translated materials into Spanish and other languages. These tangible responses to community input showed that voices were not only heard but also valued.

### Collaboration and shared accountability

Collaboration within the RCT extended beyond consultation to genuine co-ownership of processes and outcomes. Public health professionals and community partners jointly co-design summit sessions, reviewed meeting documentation, and co-authored communication materials distributed across SCC. This equitable structure fosters a culture of shared accountability, where progress is collectively defined and celebrated. Reflection became routine practice; participants used debrief activities to capture lessons learned, document challenges, and adapt strategies for upcoming sessions. These activities reinforce that co-design is a living process shaped by people engaged in it.

## Conclusion

The Co-design Conceptual Model presents valuable opportunities for public health professionals to engage in authentic, equity-driven co-design approaches. By embedding an intentional onboarding process, public health professionals are better prepared to engage community partners with an equity-driven purpose. Centering community voices allow public health professionals to ensure solutions that reflect lived experiences to address root causes of inequities in a sustainable way.

Despite these successes, the co-design process also revealed important implementation challenges. Staff and community partner turnover, particularly among key facilitators, occasionally disrupted relationship continuity and slowed momentum. Funding delays affected partner compensation and limited the ability to sustain engagement between project cycles. Additionally, the intensity of ongoing crisis response work during the COVID-19 pandemic contributed to emotional fatigue among both public health professionals and community partners. These realities underscore the need for ongoing support structures, such as peer reflection spaces, funding stability, and leadership continuity, to sustain co-design over time. Together, these perspectives reinforce the idea that co-design is an evolving practice that thrives on flexibility, reflection, and mutual accountability.

### Future directions

Looking ahead, this model provides a practical framework for transforming the way public health systems operate. As the field continues to evolve, the integration of co-design values must extend beyond specialized units and establish operations across internal departments and partner networks. A regionalized call to action can further amplify the reach of co-design by recruiting new co-designers who reflect the diversity of communities served. Expanding this work will require organizational readiness and identify workforce barriers that hinder authentic engagement. Future implementation should include formal evaluation of partner outcomes, sustained funding for CBO capacity, and integration of co-design principles across all CCDPH units. Ultimately, the co-design model invites public health professionals to reimagine traditional planning approaches and commit to partnerships rooted in equity and power-sharing. By embracing this approach, public health agencies can co-design systems that are more inclusive, sustainable, and responsive to the communities they serve.

## Data Availability

The raw data supporting the conclusions of this article will be made available by the authors, without undue reservation.

## Appendix A: RLAN co-design team principles

We hold ourselves accountable to the following principles throughout our co-design process:

1. Commit to a culture that allows people to be courageous. Everyone’s voice matters!
2. Seek to understand. Your truth is not the only truth.
3. Pursue our shared goal and use data to improve. Fail forward fast!
4. Prioritize insights from people with lived experience of inequities.
5. Practice the art of being comfortable with being uncomfortable.
6. Acknowledge we are human beings, beyond the role that we play in our jobs.
7. Acknowledge when harm has been done and mistakes have been made.
8. Be authentic: Say what you mean. Mean what you say.
9. Do what you say you are going to do. If unable, communicate it as soon as possible.
10. Address root causes of inequities and power within and across systems.
